# Soluble guanylyl cyclase α1 subunit is a key mediator of proliferation, survival, and migration in ECC-1 and HeLa cell lines

**DOI:** 10.1038/s41598-019-51420-5

**Published:** 2019-10-15

**Authors:** Sonia A. Ronchetti, María Teresa L. Pino, Georgina Cordeiro, Sabrina N. Bollani, Analía G. Ricci, Beatriz H. Duvilanski, Jimena P. Cabilla

**Affiliations:** 1 Instituto de Investigaciones Biomédicas (UBA-CONICET), Facultad de Medicina, Universidad de Buenos Aires, Ciudad Autónoma de Buenos Aires, Buenos Aires, Argentina; 2 Centro de Altos Estudios en Ciencias Humanas y de la Salud (CAECIHS), Universidad Abierta Interamericana (UAI), Ciudad Autónoma de Buenos Aires, Buenos Aires, Argentina; 3 Instituto de Biología y Medicina Experimental (IByME-CONICET), Ciudad Autónoma de Buenos Aires, Buenos Aires, Argentina

**Keywords:** Steroid hormones, RNA, Gynaecological cancer

## Abstract

Soluble guanylyl cyclase (sGC) is a heterodimeric enzyme constituted by two subunits, α1 and β1. Previously we have shown that 17β-estradiol (E2) exerts opposite effects on these subunits by increasing α1 and decreasing both β1 expression and enzymatic activity. To date, the physiological relevance of E2-induced sGC subunits’ imbalance has not been addressed. Also, increased levels strongly correlate with E2-induced proliferation in E2-dependent tissues. The aim of the present study was to investigate the role of sGCα1 in proliferation, survival, and migration in two E2-responsive and non-responsive tumour cell lines. Here we showed that E2 stimulated sGCα1 expression in ECC-1 endometrial cancer cells. sGCα1 knock-down significantly reduced E2-dependent cell proliferation. Moreover, sGCα1 silencing caused G1 arrest together with an increase in cell death and dramatically inhibited cell migration. Surprisingly, disruption of sGCα1 expression caused a similar effect even in absence of E2. Confirming this effect, sGCα1 knock-down also augmented cell death and decreased proliferation and migration in E2-unresponsive HeLa cervical cancer cells. Our results show that sGCα1 mediated cell proliferation, survival, and migration in ECC-1 and HeLa cells and suggest that sGCα1 can not only mediate E2-tumour promoting effects but can also be involved in hormone-independent tumour progression.

## Introduction

Nitric oxide-sensitive or soluble guanylyl cyclase (sGC, EC 4.6.1.2) is the main receptor of nitric oxide, which has a crucial role in signal transduction in both animals and plants^1^. Upon NO binding, sGC catalyses the formation of 3′, 5′-cyclic guanosine monophosphate (cGMP) from guanosine 5′-triphosphate. cGMP in turn activates ion channels, protein kinases and phosphodiesterases. Many of these signalling pathways have been linked to NO-induced cell death in different tissues^2–6^.

sGC, present in almost every cell type in humans^7^, is comprised of two subunits, sGCα and sGCβ, whose many isoforms differ in terms of activity, cell localization, and expression levels. The most common and abundant heterodimer is sGCα1 (GUCY1A3)/sGCβ1 (GUCY1B3) which yields maximum enzymatic activity. Although heterodimer formation is necessary for its enzyme function, sGCα1 and sGCβ1 are encoded by separate genes and are independently regulated in most human tissues^7^. Previous works from our lab have shown that E2 differentially modifies sGCα1 and sGCβ1 expression by augmenting sGCα1 and decreasing sGCβ1 in many experimental conditions (*in vivo* and *in vitro*) in anterior pituitary^8–10^. Moreover, sGCα1 expression was shown to be very sensitive to E2 or E2-like compounds such as cadmium and arsenic which mimic E2 effects in hormone-dependent tissues such as anterior pituitary and uterus^11^. The increase of sGCα1 positively correlates with E2 acute or chronic treatment and with cell proliferation whereas sGCβ1 levels diminished or were not affected^11^.

To date, there are very few cases of independent variations in subunits from the same protein. Human chorionic gonadotropin^12^, inhibin from endothelial epithelium^13^, and DNA-dependent protein kinase^14^ are examples in which the relevance of the subunits’ imbalance is almost completely unknown. The case of sGC is also fascinating as the separate roles of sGCα1 and sGCβ1 in processes unrelated to its enzymatic activity remain largely unclear and need to be exhaustively studied. Regarding sGCα1 subunit, some reports have shown its involvement in androgen-dependent cell proliferation and tumour progression in prostate cell line LNCaP^15,16^. Other supporting evidence further suggests a role of sGCα1 in tumour cell proliferation. According to the Human Protein Atlas, moderate to strong sGCα1 staining was observed in a majority of malignancies, while most cancer cells displayed weak to moderate sGCβ1 staining^17^. Conversely, the sGCβ1 role in cell fate is almost completely unknown.

Endometrial carcinoma (EC) is a major cause of morbidity and mortality for women worldwide^18^. In recent decades, the incidence of EC has been increasing in most regions of the world^19^. Clinical evidence shows that over 80% of EC corresponds to adenocarcinomas (type I) which are frequently associated with excessive estrogen exposure, mainly to 17β-estradiol (E2). In this regard, E2 is considered the principal risk factor promoting development, progression, and invasion of EC^20,21^. Identification and description of new factors involved in the progress of EC is required for the design of effective therapeutic strategies.

Cervical cancer, caused by high-risk human papillomaviruses (HPV), is one of the leading malignancies among women worldwide. E2 has been largely unrelated to its aetiology, onset, and progression, based on clinical evidence of lack of E2 receptor (ER) in biopsies from patients^22^.

Bearing in mind the importance of E2 in the progression of several gynaecological malignancies, together with our evidence of E2 upregulation of sGCα1 in hormone-dependent tissues, it was of interest to address whether sGCα1 is involved in the tumour promoting activities of E2 in an E2-dependent endometrial tumour cell line (ECC-1). The role of sGCα1 was also investigated in an E2-independent cervical tumour cell line (HeLa).

## Results

### sGCα1 expression was up-regulated by E2 in ECC-1 cells

**Figure 1:**
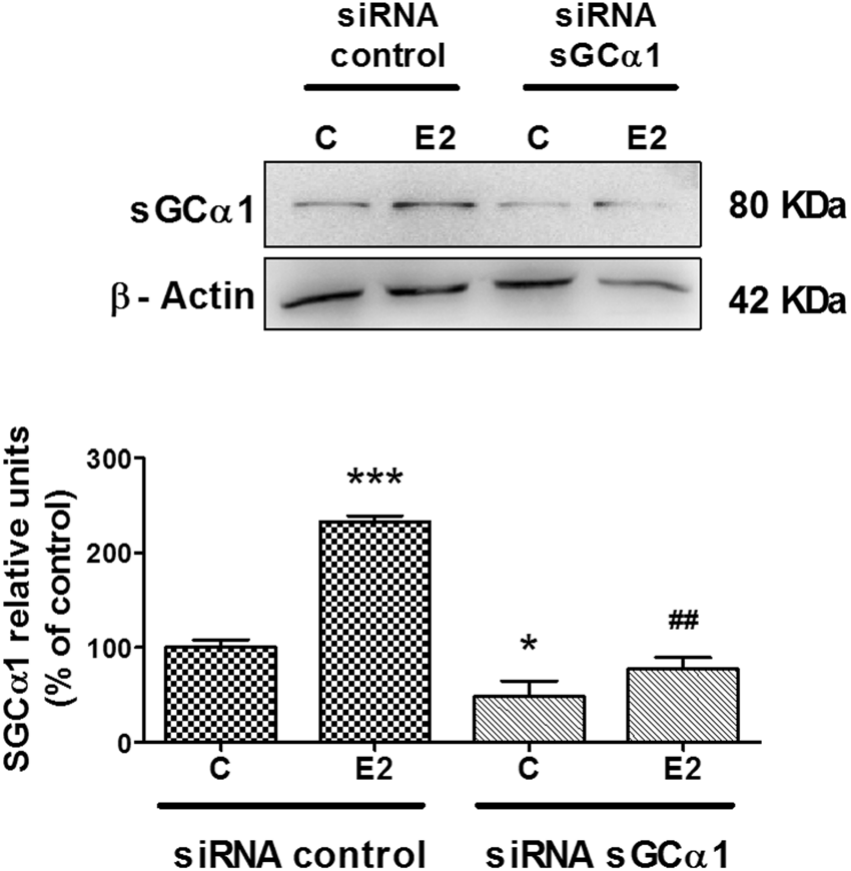
sGCα1 subunit knock-down prevented E2-induced sGCα1 increase in ECC-1 cells. Cells were transfected with sGCα1 siRNA or scramble sequences. Six h post-silencing, cells were incubated with or without 1 nM E2 for 48 h. sGCα1 protein expression was determined by western blot. Representative western blots are from the same gel in their original order and are shown after cropping, aligning, and separating them by white space. Raw data are presented in Supplementary Dataset. Results are shown as mean ± SE of average densitometric values of sGCα1 relative to β-actin. ANOVA followed by Tukey’s test, *p < 0.05; ***p < 0.001 vs. siRNA control; ^##^p < 0.01 vs. siRNA control + E2 (N = 3).

To address the role of sGCα1 in cell proliferation, we employed an experimental design in which we disrupted sGCα1 subunit levels through siRNA-directed downregulation of sGCα1 expression. ECC-1 cells were transfected with sGCα1-targeted siRNA oligonucleotides which resulted in a significant reduction of sGCα1 protein levels (Fig. 1). Scrambled sequences used as controls had no effect on sGCα1 expression compared to non-transfected cells or cells treated with lipofectamine alone, thereby verifying the specificity of the siRNA sequences (Supplementary Figure S1a).

Then, to address the effect of E2 on sGCα1 subunit levels, ECC-1 cells were incubated with 1 nM E2 for 48 h, a concentration 10 times lower than the one usually employed^23^. As expected, E2 markedly augmented sGCα1 protein levels. sGCα1 knock-down was shown to significantly reduce sGCα1 expression. Moreover, sGCα1 silencing was effective even under E2 stimulating effects (Fig. 1). These results show that sGCα1 expression was augmented by E2 in ECC-1 cells, concording with previous reports on other E2-dependent tissues^8,11^. These results also confirmed that the stimulatory effect of E2 on sGCα1 was mainly transcriptional as formerly described^10^, previous to post-transcriptional sGCα1 degradation by RNA-induced silencing complexes (RISC).

### sGCα1 was directly involved in ECC-1 cell proliferation

**Figure 2:**
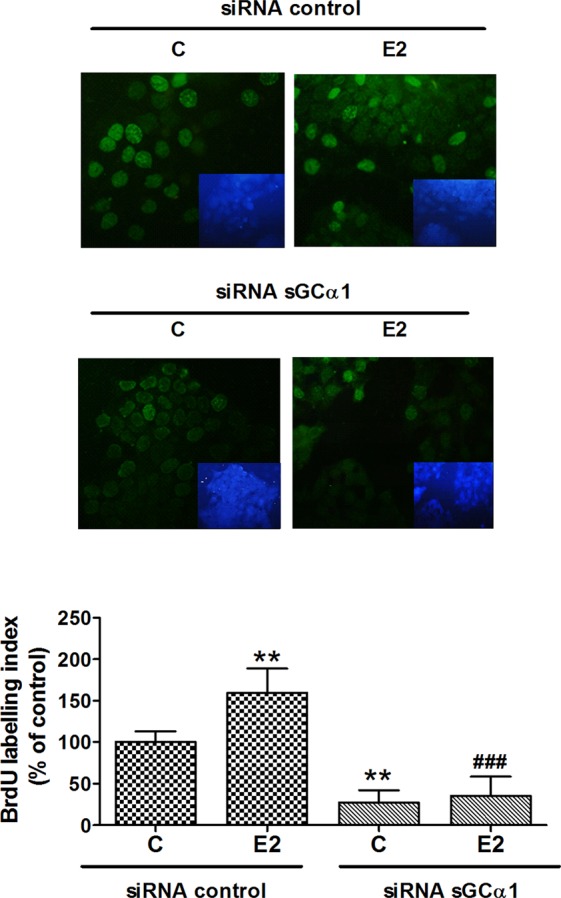
sGCα1 subunit knock-down reduced ECC-1 cell proliferation. Cells were transfected with sGCα1 siRNA or scramble sequences. Six h post-silencing, cells were incubated with or without 1 nM E2 for 48 h. Cell growth was assessed by BrdU incorporation. Bars represent the BrdU labelling index expressed as BrdU-positive nuclei/total cell nuclei × 100. Magnification, 40X. ANOVA followed by Tukey’s test, **p < 0.01 vs. siRNA control; ^###^p < 0.001 vs. siRNA control + E2 (N = 3).

To address the importance of sGCα1 in cellular proliferation, we evaluated BrdU incorporation to DNA. 1 nM E2 treatment significantly increased the number of BrdU-positive cells, thereby indicating that this E2 concentration was enough to induce ECC-1 cell proliferation. sGCα1 knock-down was shown to be associated with a strong decrease in BrdU labelling index in both presence and absence of 1 nM E2 treatment (Fig. 2).

To further confirm the involvement of sGCα1 in cell proliferation, we studied the expression of three classic protein markers of cell cycle progression by western blot: proliferating cell nuclear antigen (PCNA), which is constitutively expressed throughout the cell cycle in actively growing cells, and cyclin D1 (CCND1) and cyclin E (CCNE) which control G1/S transition.

**Figure 3:**
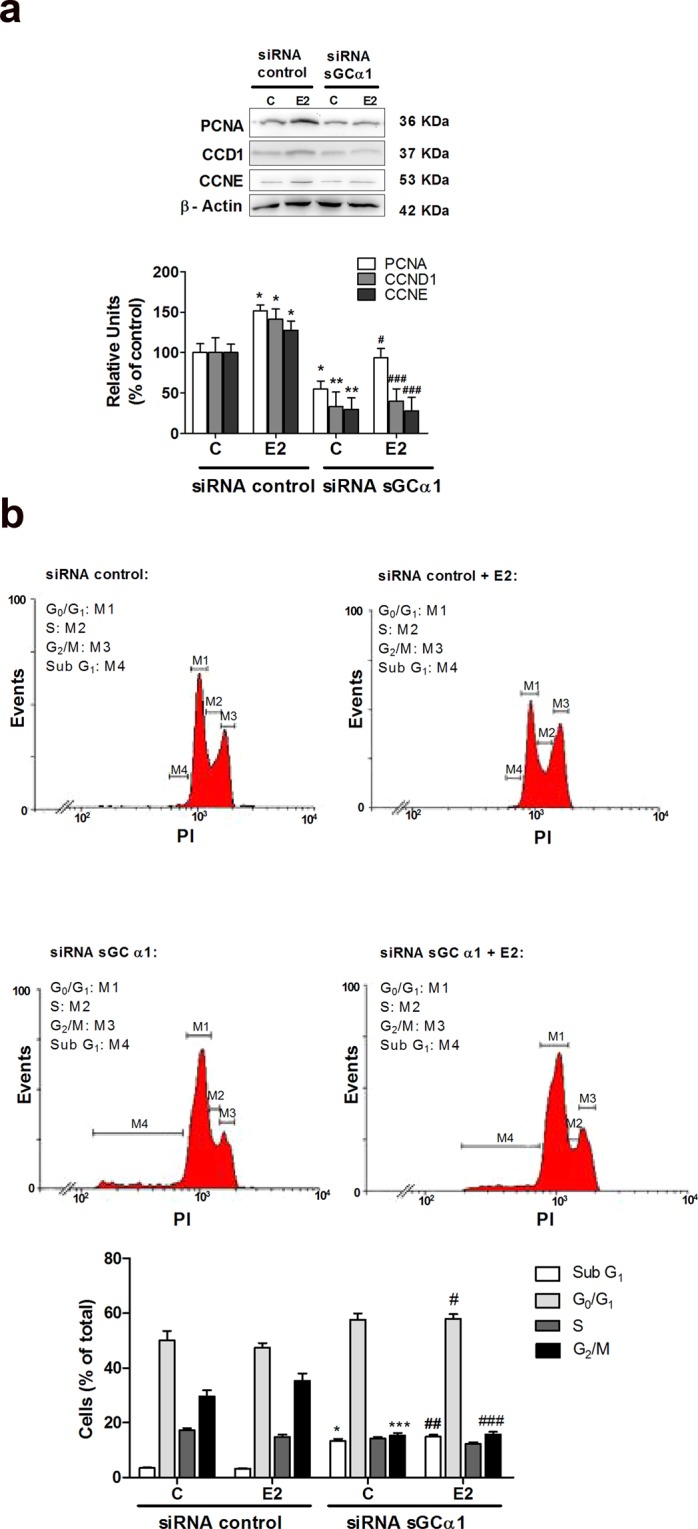
sGCα1 subunit silencing down-regulated cell proliferation marker levels, affected cell cycle distribution and increased hypodiploidy. Cells were transfected with sGCα1 siRNA or scramble sequences. Six h post-silencing, cells were incubated with or without 1 nM E2 for 48 h. (**a**) PCNA, cyclin D1 (CCND1) and cyclin E (CCNE) protein expression was evaluated by western blot. Representative western blots are from the same gel in their original order and are shown after cropping, aligning, and separating them by white space. Raw data are presented in Supplementary Dataset. Bars represent mean ± SE of average densitometric values of PCNA, CCND1 or CCNE relative to β-actin. ANOVA followed by Tukey’s test, *p < 0.05, **p < 0.01 vs. siRNA control; ^#^p < 0.05, ^###^p < 0.001 vs. siRNA control + E2 (N = 3). (**b**) Cells were stained with propidium iodide and analysed by flow cytometry. Histograms represent three independent experiments. ANOVA followed by Tukey’s test, *p < 0.05, ***p < 0.001 vs. same cell cycle phase from siRNA control; ^#^p < 0.05, ^##^p < 0.01, ^###^p < 0.001 vs. same cell cycle phase from siRNA control + E2 (N = 3).

As expected, 1 nM E2 significantly increased the expression of all cell cycle positive regulators analysed. sGCα1 silencing significantly reduced the expression of PCNA, CCND1 and CCNE either in presence or absence of E2 stimulus (Fig. 3a).

Overall, these results provide the first direct evidence of sGCα1 role in E2-dependent and independent cell proliferation by modifying the expression of cell cycle progression markers in endometrial cancer cells.

### sGCα1 was involved in cell cycle progression and death

Bearing in mind previous results, we aimed to study the effects of sGCα1 silencing on cell cycle progression. Cell cycle distribution of propidium iodide-labelled cells was analysed. As expected, 1 nM E2 treatment produced a decrease in G1 phase together with an increase of G2/M phases compared to untreated cells. In sGCα1-silenced cells, an increase in G0/G1 DNA content together with a decrease in the percentage of cell DNA in phase S and G2/M was observed both presence and absence of E2 stimulus, compared to respective controls (Fig. 3b). Collectively, these data suggest that sGCα1 knock-down could lead to an arrest of cells in G0/G1 phase, a delayed transition into S phase, and eventually, restrained proliferation of cells.

Unexpectedly, sGCα1 silencing augmented the cell population with reduced DNA content (sub G1 phase) (Fig. 3b), suggesting that sGCα1 could also be involved in cell survival.

**Figure 4:**
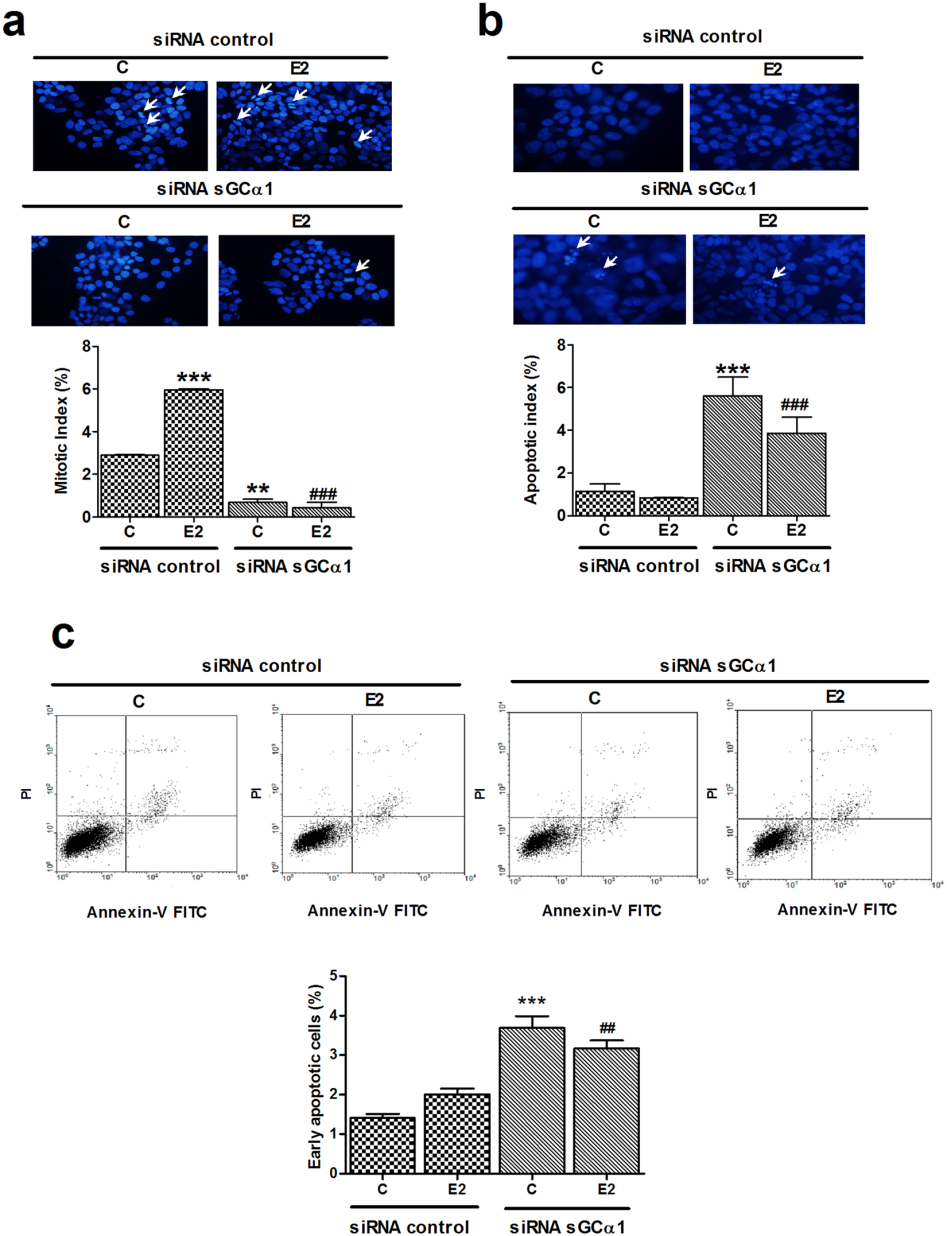
sGCα1 silencing decreased mitotic index and augmented apoptosis. Cells were transfected with sGCα1 siRNA or scramble sequences. Six h post-silencing, cells were incubated or not with 1 nM E2 for 48 h. Cells were fixed and stained with Hoechst 32258 for nuclear morphology study. Representative images obtained at 40X. Bars represent the mean ± SE of mitotic (**a**) and apoptotic (**b**) indices, expressed as percentage of total cell number. Arrows point to mitotic (**a**) and apoptotic (**b**) nuclei. (**c**) Cells were stained with annexin-V FITC and propidium iodide (PI) and analysed by flow cytometry. Annexin-V-positive, PI-negative cells were considered early apoptotic cells, and those double stained, late apoptotic or necrotic cells. Dot plots represent three independent experiments. ANOVA followed by Tukey’s test, **p < 0.01, ***p < 0.001 vs. siRNA control; ^##^p < 0.01, ^###^p < 0.001 vs. siRNA control + E2, (N = 3).

To further confirm sGCα1 silencing-driven cell death, cells were stained with Hoechst 32258 and nuclear morphology was analysed. Concordantly with previous results, 1 nM E2 treatment significantly increased mitotic index (Fig. 4a) without modifying apoptotic index (Fig. 4b). sGCα1 knock-down cells showed a significant increase in apoptotic index (Fig. 4b), even in presence of pro-mitotic E2 stimulus. In line with results obtained by flow cytometry and proliferation marker levels, a lower mitotic index was observed in sGCα1-silenced cells both in presence and absence of E2 compared to respective controls (Fig. 4a). In order to prove that the disruption of sGCα1 expression led to apoptosis, cells were double-stained with annexin-V FITC and PI and analysed by flow cytometry. sGCα1 silencing augmented the percentage of both early apoptotic (Annexin-V positive/PI-negative) and late apoptotic/necrotic (positive double staining) cell populations. In concordance with previous results, sGCα1 knock-down increased the percentage of cell death even in presence of E2 (Fig. 4c).

All together, these results suggest that sGCα1 plays a key role in cell proliferation as well as in cell survival.

### sGCα1 silencing reduced ECC-1 cell migration

**Figure 5:**
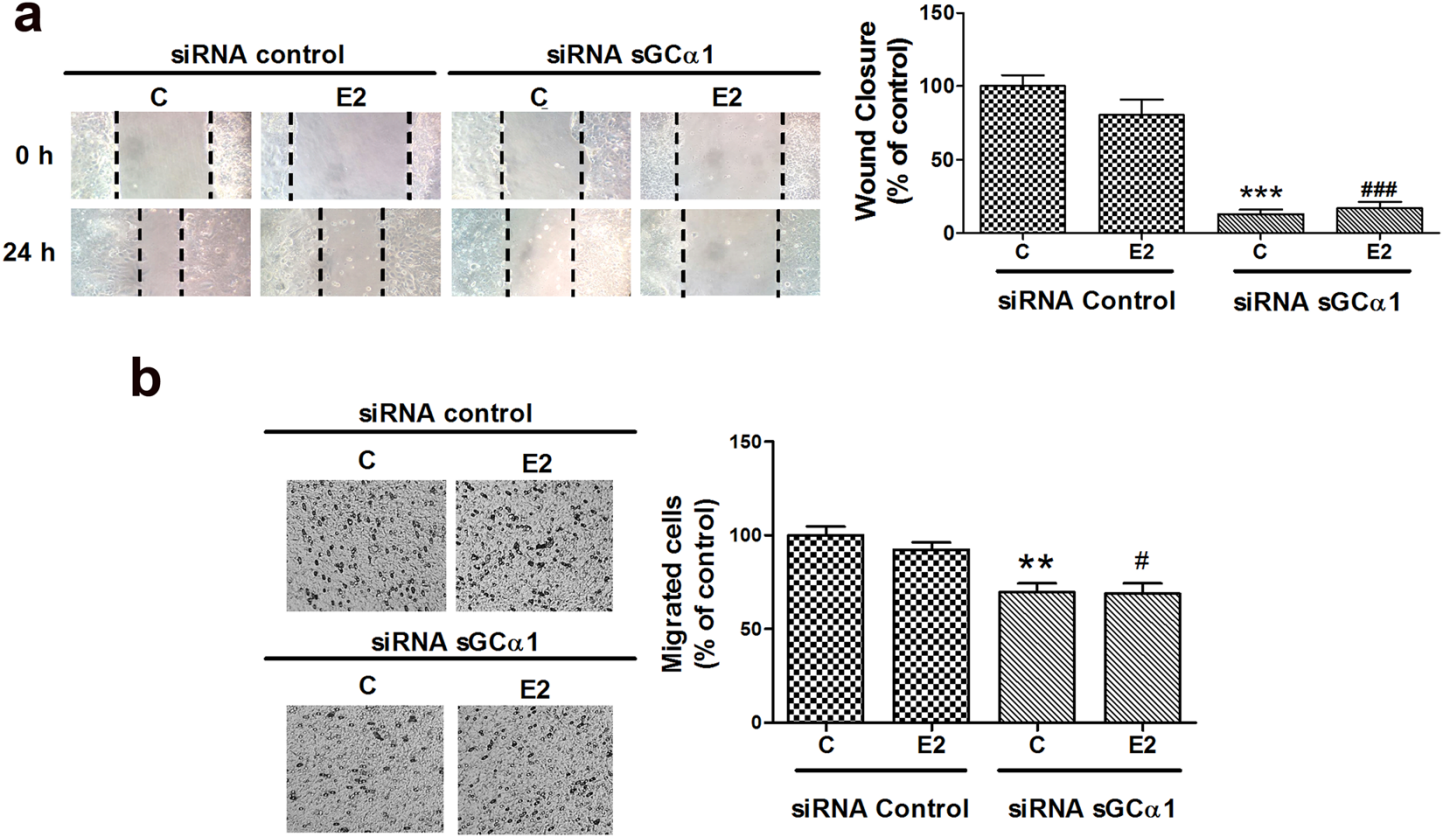
sGCα1 knock-down reduced cell migration. Cells were transfected with sGCα1 siRNA or scramble sequences. Six h post-silencing, cells were incubated or not with 1 nM E2 for 24 h and cell migration was assessed by scratch wound (**a**) and transwell migration (**b**) assays. Representative images were obtained at 40X. Bars represent the relative wound closure area after 24 h incubation expressed as percent of control (**a**) or migrated cells after 24 h as percent of control (**b**). ANOVA followed by Tukey’s test, **p < 0.01, ***p < 0.001 vs. siRNA control; ^#^p < 0.05, ^###^p < 0.001 vs siRNA control + E2 (N = 3).

Migration of cancer cells away from the primary tumour is one of the earliest events in metastatic process^24^. In order to evaluate whether sGCα1 is involved in cell migration, scratch motility and transwell migration assays were performed. 1 nM E2 treatment showed no significant difference in migration compared to control, but sGCα1 knock-down dramatically reduced cell migration in both presence and absence of E2 (Fig. 5a,b). These results suggest that sGCα1 is involved not only in cell proliferation and survival but also in cell migration.

### sGCα1 mediated cell proliferation, survival, and migration of E2-nonresponsive HeLa cells

In contrast to ECC-1 cells, HeLa cells – like most cervical cancer cells - are ER negative. Consequently, we wanted to analyse whether sGCα1 participates in cell growth and fate in this cell line, in absence of E2 stimulus.

**Figure 6:**
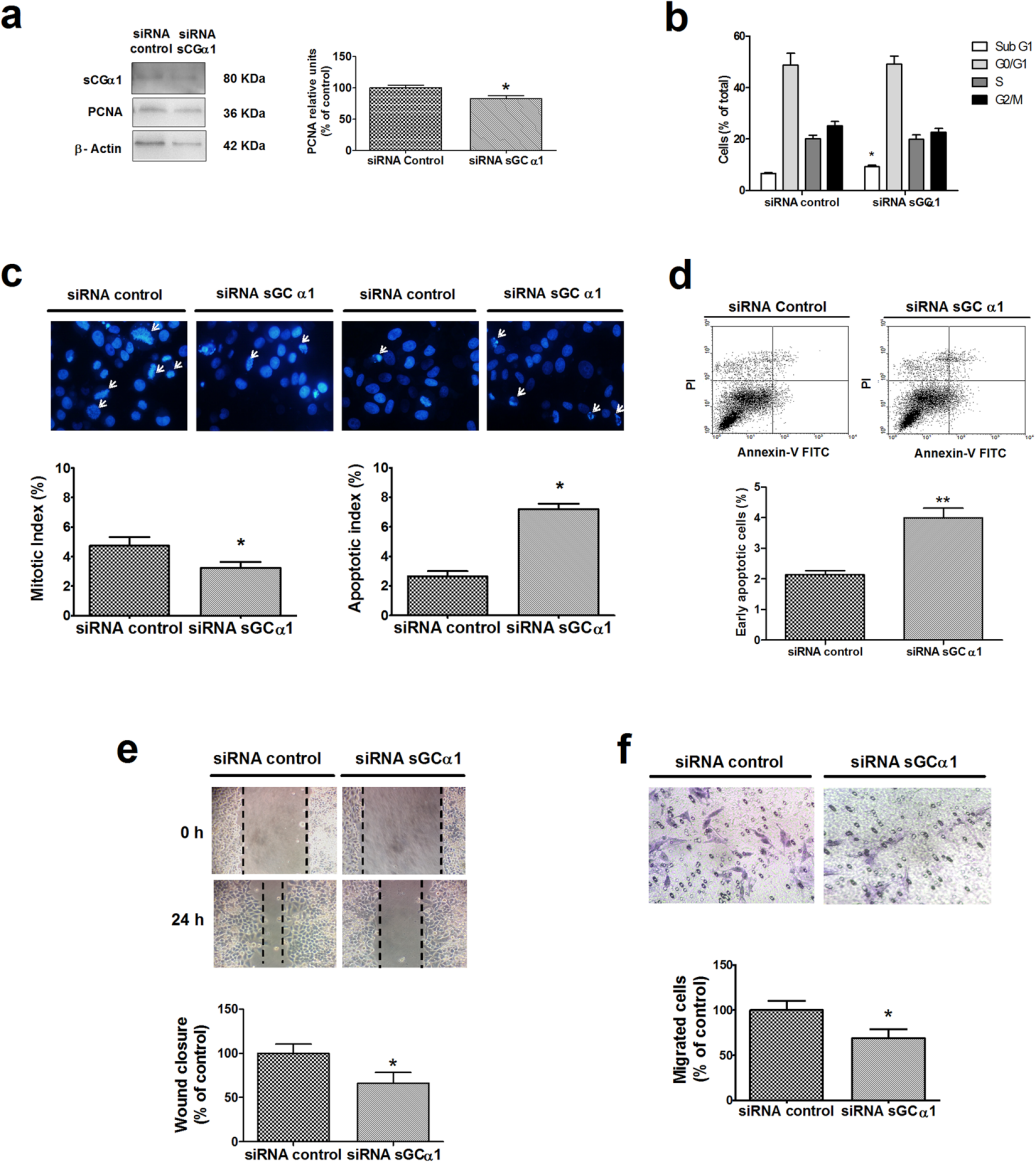
sGCα1 silencing reduced PCNA expression, cell survival, and migration in HeLa cells. Cells were transfected with sGCα1 siRNA or scramble sequences (control). After 6 h, media was replaced by fresh media and cells were incubated for 48 h. (**a**) PCNA levels were determined by western blot. Bars represent mean ± SE of average densitometric values of PCNA relative to β-actin. Representative western blots are from the same gel in their original order and are shown after cropping, aligning and separating them by white space. Raw data are presented in Supplementary Dataset. (**b**) Cells were stained with propidium iodide and analysed by flow cytometry. (**c**) Cells were fixed and stained with Hoechst 32258 for nuclear morphology study. Representative images obtained at 40X. Bars represent mean ± SE of mitotic or apoptotic indices, expressed as percent of total cell number. (**d**) Cells were stained with annexin-V FITC and propidium iodide (PI) and analysed by flow cytometry. Annexin-V-positive, PI-negative cells were considered early apoptotic cells, and those double stained, late apoptotic or necrotic cells. Dot plots represent three independent experiments. (**e,f**) Cell migration was assessed by scratch wound (**e**) and transwell (**f**) assays. Representative images were obtained at 40X. Bars represent the relative wound closure area after 24 h incubation (**e**) or migrated cells after 24 h (**f**), as percent of control. Student’s ‘*t*’ test, *p < 0.05, **p < 0.01 (N = 3).

sGCα1 is expressed in HeLa cell line in very much lower levels than those found in ECC-1 cells. Scrambled sequences used as controls had no effect on sGCα1 expression compared to non-transfected cells or cells treated with lipofectamine alone (Supplementary Figure S1b). However, sGCα1 silencing was effective since it down-regulated sGCα1 levels (1.3-fold decrease, p < 0.05) and significantly reduced PCNA levels (Fig. 6a). Unlike ECC-1 cells, cell cycle distribution profiles showed no differences between the two conditions, but subG1 population was 41% higher in sGCα1-silenced cells (Fig. 6b). This augment of hypodiploidy was further confirmed by nuclear morphology analysis, showing a significant increase of apoptotic index together with a reduction of mitotic index (Fig. 6c). Moreover, flow cytometry analysis showed that sGCα1 knock-down increased Annexin-V-positive cell population, indicative of apoptotic cells (Fig. 6d). In line with observations of ECC-1 cells, these results suggest that sGCα1 is also involved in HeLa cell survival.

Cell migration was also affected by sGCα1 knock-down. 24 h after wounding, sGCα1 silenced cells presented significantly lower wound recovery than the control group (Fig. 6e). In a similar way, sGCα1 knock-down diminished the number of cells that crossed the polycarbonate membrane assessed by transwell assay (Fig. 6f).

All together, these results suggest that sGCα1 is also a novel factor modulating cell proliferation and progression unrelated to E2 signalling.

## Discussion

A large body of evidence supports the role of E2 in the onset and progression of multiple cancers. In this regard, classical and nonclassical hormone-sensitive carcinomas including breast, endometrial, ovarian, colon, prostate, and lung have been strongly related to the oncogenic role of E2^25^.

The evidence showing that E2 causes an imbalance in sGC subunit expression^8–10^ and that sGCα1 levels directly correlate with E2-induced cell proliferation^11^ led us to investigate sGCα1 as a potential factor mediating E2 pro-tumoural effects in an endometrial cancer cell line.

Our results showed that sGCα1 expression increased after E2 treatment in ECC-1 cells. This finding tallies with previous reports from our lab showing that sGCα1 levels augment in response to chronic or acute E2 treatment in different hormone-dependent tissues such as pituitary gland and uterus^8,11^. Moreover, we demonstrated that sGCα1 is highly sensitive to a wide range of endocrine disruptors with xenoestrogenic activity such as some pesticides, organochlorine compounds, synthetic hormones (unpublished data) and heavy metals^11^.

The rise in sGCα1 levels correlated with cell proliferation in these E2-responsive tissues^11^. This finding concords with another, reported for prostate cell lines, where androgen-driven sGCα1 increase directly correlates with cell proliferation^15^. Based on this evidence, we addressed whether sGCα1 was involved in cell proliferation in a well-known, E2-dependent endometrial cancer cell line.

Here we report that sGCα1 knock-down caused G1 arrest which led to a G1/S transition delay and therefore a reduction of cell cycle progression marker expression such as PCNA, CCND1 and CCNE. This was further confirmed by a reduction of both DNA-replicating cells and mitotic nuclei. Remarkably, this was observed both presence and absence of E2 stimulus, which underscores that sGCα1 may be an important mediator not only of E2 proliferative actions but also of other, non E2-related proliferation pathways.

As we expected, sGCα1 knock-down increased the percentage of hypodiploid cells -indicative of cell death- probably due to G1 arrest, confirmed by increased apoptotic nuclei morphology and annexin-V-positive cells. Our results showing that some key cell cycle markers of G1/S transition were affected after sGCα1 silencing suggest that proliferative effects of sGCα1 likely take place at this point or earlier. sGCα1 has recently been shown to sequestrate p53 into the cytoplasm thereby inhibiting its transcriptional activity in prostate cell lines^26^. ECC-1 and other endometrial cancer cell lines express functional p53^27^. Considering that p53 is a critical cell cycle checkpoint protein governing progression of cells from G1 into S phase, it seems probable that G1 arrest and therefore cell death of ECC-1 cells may be a consequence of normal p53 activity in the absence of the strong inhibitory action of sGCα1. This hypothesis is currently under investigation in our lab.

Here we show for the first time that sGCα1 was also involved in one of the most important steps in metastatic dissemination: cell migration, which was observed by wound healing and transwell migration assays. Previous reports demonstrated that 10 nM E2 treatment induced endometrial cancer cell migration^23^. In the present study, we used an E2 concentration 10 times lower than the one usually employed in this cell line. We chose this dose since a higher E2 dose could exert stronger stimulation of sGCα1 transcription^9^ and consequently interfere with sGCα1 silencing experiments. Remarkably, we found that 1 nM E2 exerts significant proliferative effects but is unable to increase cell migration, probably because this E2 concentration is insufficient to fully trigger cell migration mechanisms. In the present work we demonstrated that sGCα1 knock-down inhibited cell migration in absence of E2 stimulus, again suggesting that sGCα1 can play a role in hormone-independent tumour progression.

Since evidence provided by our studies and others indicates that sGCα1 pro-tumoural actions are linked to hormone stimulus and sGCα1-driven p53 inactivation, we decided to investigate the role of sGCα1 in HeLa, the E2 non-responsive, p53-defective cell line.

Here, we demonstrated that sGCα1 knock-down notably affected cell proliferation, evidenced by a reduction of PCNA expression and mitotic index. As observed in ECC-1 cells, disruption of sGCα1 expression caused increased cell death mainly by apoptosis. Additionally, sGCα1-silenced HeLa cells were shown to be less aggressive since migratory capability was strongly affected. Collectively, this evidence broadens the perspective not only on hormone-dependent cancers but also on the non-dependents or their hormone-refractory counterparts and those with early loss of p53. These scenarios need to be exhaustively studied.

In line with our findings, Mohammadoo-Khorasani *et al*. recently showed that of a total of 105 human breast biopsies, sGCα1 protein expression was higher in all malignant breast tumours than in those of benign or normal tissues, independent of hormone receptor status or tumour stage^28^.

In sum, this work provides new evidence of sGCα1 subunit in a completely different role. Here we show that sGCα1 was a crucial factor in cell proliferation, migration, and survival in both E2-responsive and non-responsive cancer. The fact that sGCα1 also exerts tumour-promoting effects in absence of E2 suggests that this subunit may also be involved in hormone-independent tumour progression. Therefore, sGCα1 subunit must be intensively investigated as a novel, promising therapeutic target in both hormone-dependent and independent tumours.

## Materials and Methods

### Cell cultures

ECC-1 is a well-differentiated human endometrial epithelial cell line responsive to sex hormones^29–33^. HeLa is an HPV-infected human cervical cancer cell line non-responsive to E2^34–36^. ECC-1 and HeLa cell lines were purchased from ATCC (Manassas, VA) and generously provided by Laboratorio de Inmunología de la Reproducción, Instituto de Biología y Medicina Experimental (IByME-CONICET), and by Dr. Viviana Blank (Instituto of Química y Físicoquímica Biológicas, Facultad de Farmacia y Bioquímica, Universidad de Buenos Aires), respectively. Experiments were conducted within one year of their purchase from ATCC. Cells were grown in Roswell Park Memorial Institute (RPMI) media (Gibco, Waltham, MA, USA) supplemented with 10% fetal bovine serum (GenSa, Buenos Aires, Argentina) and penicillin-streptomycin mixture (50 units/mL and 50 μg/mL) and kept at 37 °C and 5% CO_2_. Since ECC-1 cells were shown to be very sensitive to charcoal-stripped serum conditions, all experiments were performed in complete RPMI supplemented with 5% fetal bovine serum. Control of each experiment was performed with the same culture media. Experiments in HeLa cells were performed in the same conditions.

### siRNA sequences and transfection

The sequence of sGCα1 used to design siRNA was accession number NM_017090. Sequences of the RNA oligonucleotides, designed by BLOCK-iT™ RNAi Designer algorithm, available online: (http://rnaidesigner.invitrogen.com/rnaiexpress/) (Invitrogen, Carlsbad, USA), are described in Table 1.

**Table 1:** siRNA sequences used for silencing sGCα1.

Target mRNA	siRNA primer sequence	Start site
sGC α1	5′ CACCUGCCAGGAGUUUGCUAGAAU 3′	272
	5′ AAGACUCUCUGGGUGAGGAACUGUU 3′	532
	5′ AGGACCAGGACUUUCUAAAUGUUUA 3′	718
Scramble	UAACUGCUGAUUUGGGUGGGUGGCCACAC	N/A
	AACAGUUCCUCACCCAGAGAGUCUU	N/A
	UAAACAUUUAGAAAGUCCUGGUCCU	N/A

ECC-1 and HeLa cells (7.10^4^ per well) were seeded on 24-well plates and transfected with siRNAs using Lipofectamine^TM^ 2000 (Invitrogen) according to manufacturer’s instructions. Briefly, 1 μL of lipofectamine, incubated in 50 μL of Opti-MEM (Invitrogen) for 5 min, was added to an equal volume of Opti-MEM containing siRNA pool (final concentration: 50 nM). Mixture was incubated for 20 min and 100 µL of this mixture were added to each well containing 400 µL of antibiotic-free media. After 6 h incubation, culture media was removed and replaced with complete medium. Cells transfected with scramble sequences showed no visible alteration in morphology, growth, or viability, and no differences compared to cells transfected with lipofectamine alone or untreated cells (Supplementary Figure S2).

### Western blot analysis

This technique was performed as previously described^11^. Briefly, about 40 μg of total protein from each sample were boiled for 5 min in Laemmli sample buffer and fractionated on 10 or 12% SDS–PAGE. Resolved proteins were transferred to polyvinylidene difluoride membranes and blocked for 24 h at 4 °C in blocking buffer (5% nonfat dry milk in 1% T-TBS). Then, membranes were co-incubated overnight at 4 °C with primary antibodies: anti-cyclin E (1:500, sc-377100), anti-cyclin D1 (1:500, sc-753), anti-PCNA (1:1000, sc-56), all from Santa Cruz Biotechnology (Dallas, TX, USA); anti-sGCα1 (1:1500, G4280), and anti-β-actin (1:1000, A2066) (Sigma-Aldrich, Saint Louis, MO, USA), respectively, in blocking buffer. Blots were washed and incubated for 1 h at room temperature (RT) with secondary antibodies (1:2000), used depending on the primary antibodies: goat anti-rabbit IgG-horseradish peroxidase (Jackson, West Grove, PA, USA) and goat anti-mouse IgG-horseradish peroxidase (Sigma-Aldrich). Bands were detected using ECL detection kit (Kalium, Buenos Aires, Argentina).

### BrdU assay

Experiments were performed as previously reported^37^. Briefly, ECC-1 cells were incubated with 100 μM BrdU 3 h before the end of treatment and then fixed in 4% formaldehyde for 30 min at 4 °C, permeabilized with 6N HCl in 1% Triton X-100 in PBS for 15 min at RT and neutralized with 0.1 M sodium borate in 1% Triton X-100 in PBS for 15 min at RT. Cells were incubated in blocking solution (5% normal serum in 0.2% Triton X-100) for 2 h at RT. Cells were incubated with mouse anti-BrdU primary antibody (1:200, Sigma-Aldrich) overnight at 4 °C, and after three washes the secondary antibody conjugated to fluorescein (1:250, Sigma-Aldrich) was added. Cells were mounted in anti-fade solution containing Hoechst 32258 and DABCO. Cells were quantified by three independent blinded observers in an Olympus BX50 (Japan) fluorescence microscope. Data of at least 300 nuclei per triplicate obtained from random fields and from three independent experiments were expressed as number of BrdU labelled cells/total cell number × 100.

### Nuclear morphology analysis

Experiments were carried out as previously reported^38^. Briefly, 2.10^4^ cells were seeded onto 12 mm diameter glass coverslips on 24-well plates. After treatments, cells were fixed with 4% formaldehyde for 30 min at 4 °C and mounted in anti-fade solution containing Hoechst 32258. Nuclear morphology was observed and quantified in an Olympus BX50 (Japan) fluorescence microscope. A total amount of 1000 cells were counted per triplicate by three independent blinded observers. Apoptotic and mitotic indices were calculated as: number of apoptotic or mitotic nuclei/total number of nuclei × 100.

### Cell cycle analysis by flow cytometry

This technique was adapted from one previously described^39^. After treatment, cells were collected by trypsinization, fixed with 70% ice-cold ethanol in PBS, centrifuged, and resuspended in 0.2 mL of propidium iodide (PI) staining solution (50 μg/mL PI in PBS containing 0.2 mg/mL of DNase-free RNase A). After incubation for 30 min at 37 °C, samples were evaluated by flow cytometry with a Becton Dickinson FACScalibur flow cytometer (San Jose, CA, USA). Cell population selected by gating and cell cycle distribution was analysed using WinMDI 2.8 software (http://facs.scripps.edu).

### Apoptosis determination by annexin V–fluorescein isothiocyanate and propidium iodide staining

Experiments were performed as previously reported^40^. Briefly, the annexin V–fluorescein isothiocyanate (FITC) apoptosis detection kit (Sigma-Aldrich) was used for apoptosis determination. After treatment, cells were trypsinized and centrifuged at 1,000 × *g* for 10 minutes. Cell pellets (2.5 × 10^5^ cells) were resuspended in 250 µL annexin binding buffer followed by addition of 5 µL FITC-conjugated annexin V (exλ: 488 nm, emλ: 535 nm, FL1) and 10 µL propidium iodide (PI; exλ: 488 nm, emλ: 585 nm, FL2) solution (100 μg/mL). Cells were incubated for 15 minutes in darkness at RT and were analysed by flow cytometry (Becton Dickinson FACScalibur). At least 2.10^4^ events were measured for each treatment. Further flow cytometric data analysis was performed with WinMDI 2.8 software.

### Scratch wound assay

7.10^4^ cells were plated in a 24-well plate and grown in RPMI complete media. Then cells were silenced with siRNA sGCα1 or scramble sequences (control). The monolayer was wounded with a pipette tip and detached cells were removed after washing with PBS. Then, the scratched area was photographed at 0 and 24 h. The scratch area in each well was evaluated by ImageJ software (National Institutes of Health, USA). Wound closure was calculated as (area 0 h/area 24 h)/area 0 h and was expressed as percentage of control.

### Transwell migration assay

Cell migration was performed in an 8 µm-pore size Boyden chamber (BD Biosciences, San José, CA, USA). Control and sGCα1-silenced cells were tripsinized and a cell suspension of 2.5.10^5^ cells/mL in serum-free media was prepared. 200 µL of this suspension was added to the upper chamber of each of the transwell inserts. RPMI with 10% FBS was added to the bottom chamber as chemoattractant and incubated for 24 h. Non-migrated cells were gently swabbed from the upper chamber. Migrated cells were fixed with ice-cold methanol, stained with Giemsa, and counted under a light microscope.

### Statistical analysis

Results were expressed as mean ± SE and evaluated by Student’s ‘*t*’ test or two-way analysis of variance (ANOVA) followed by Tukey’s test. A probability value of *P* < 0.05 was considered statistically significant. All statistical analyses were run using GraphPad Prism 5.00 for Windows Graphpad Software (San Diego, CA, USA). Results were confirmed by at least three independent experiments.

### Accession codes

Soluble guanylyl cyclase alpha1 subunit (GUCY1A3) accession number: NM_017090.

## Supplementary information

Supplementary figures

Supplementary Dataset 1

## Data Availability

The datasets analysed during the current study are available from the corresponding author on reasonable request.
