# Supplementary material for: Soluble guanylyl cyclase α1 subunit is a key mediator of proliferation, survival, and migration in ECC-1 and HeLa cell lines
Source: Sci Rep. 2019 Oct 15;9:14797. doi: 10.1038/s41598-019-51420-5 (PMC6794259; doi:10.1038/s41598-019-51420-5)
Supplement: Supplementary file 1 — Supplementary figures [file 41598_2019_51420_MOESM1_ESM.docx]

**Soluble guanylyl cyclase α1 subunit is a key mediator of proliferation, survival and migration in ECC-1 and HeLa cell lines**

**Sonia A. Ronchetti**^1,2+^, **María Teresa L. Pino**^1,2+^, **Georgina Cordeiro**^1,2^_,_ **Sabrina N. Bollani**^1,2^, **Analía G. Ricci**^3^, **Beatriz H. Duvilanski**^1,2^, **Jimena P. Cabilla**^1,2^*

^1^Instituto de Investigaciones Biomédicas (UBA-CONICET), Facultad de Medicina, Universidad de Buenos Aires, Ciudad Autónoma de Buenos Aires, Argentina

^2^Centro de Altos Estudios en Ciencias Humanas y de la Salud (CAECIHS), Universidad Abierta Interamericana (UAI), Ciudad Autónoma de Buenos Aires, Argentina

^3^Instituto de Biología y Medicina Experimental (IByME-CONICET), Ciudad Autónoma de Buenos Aires, Argentina

*E-mail: jimenacabilla@hotmail.com

^+^These authors contributed equally to this work


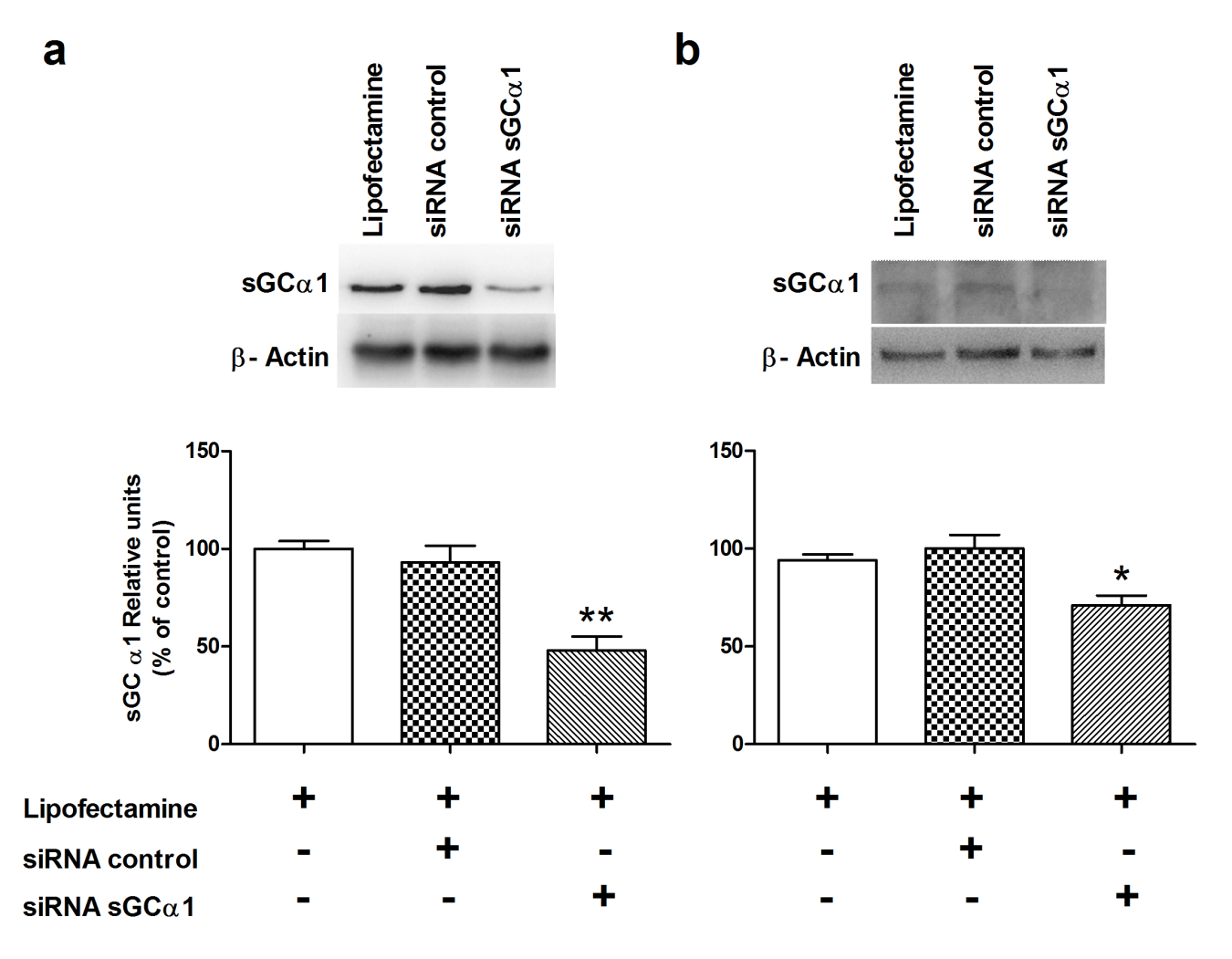


**Figure S1.** ECC-1 (**a**) and HeLa (**b**) cells were transfected for 48 h with 50 nM siRNA pool against sGCα1 subunit or with scramble sequences. sGCα1 protein expression was determined by western blot. Bars represent mean ± SE of average densitometric values of sGCα1 relative to β-actin. ANOVA followed by Tukey’s test, *p<0.05; **p<0.01 vs. siRNA control (N=3).


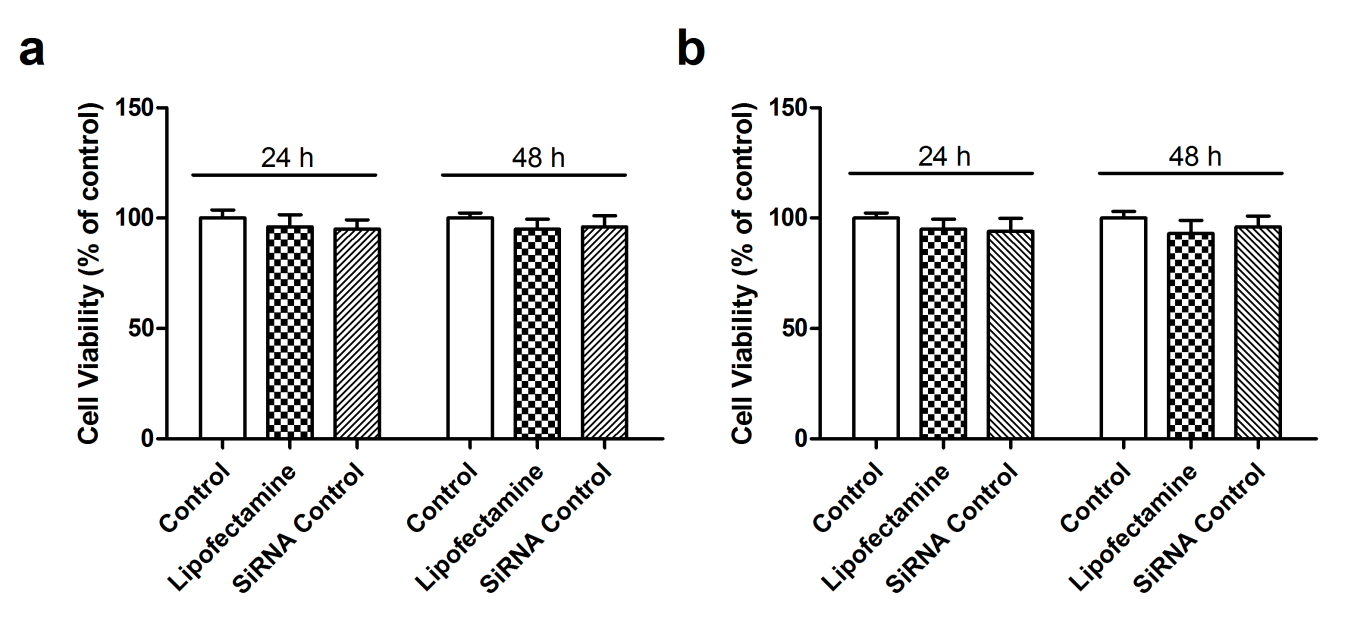


**Figure S2**. ECC-1 (**a**) and HeLa (**b**) cells were incubated with lipofectamine with or without siRNA control sequences for 6 h. Cell viability was assessed by MTT assay at 24 h and 48 h. Data represent mean ± SE of average absorbance values at 595 nm and are expressed as percent of control. ANOVA, ns. (N=3).
