# Supplementary material for: Soluble guanylyl cyclase α1 subunit is a key mediator of proliferation, survival, and migration in ECC-1 and HeLa cell lines
Source: Sci Rep. 2019 Oct 15;9:14797. doi: 10.1038/s41598-019-51420-5 (PMC6794259; doi:10.1038/s41598-019-51420-5)
Supplement: Supplementary file 2 — Supplementary Dataset 1 [file 41598_2019_51420_MOESM2_ESM.doc]

**Soluble guanylyl cyclase α1 subunit is a key mediator of proliferation, survival and migration in ECC-1 and HeLa cell lines**

**Sonia A. Ronchetti**^1,2+^, **María Teresa L. Pino**^1,2+^, **Georgina Cordeiro**^1,2^, **Sabrina N. Bollani**^1,2^, **Analía G. Ricci**^3^, **Beatriz H. Duvilanski**^1,2^, **Jimena P. Cabilla**^1,2^*

^1^Instituto de Investigaciones Biomédicas (UBA-CONICET), Facultad de Medicina, Universidad de Buenos Aires, Ciudad Autónoma de Buenos Aires, Argentina

^2^Centro de Altos Estudios en Ciencias Humanas y de la Salud (CAECIHS), Universidad Abierta Interamericana (UAI), Ciudad Autónoma de Buenos Aires, Argentina

^3^Instituto de Biología y Medicina Experimental (IByME-CONICET), Ciudad Autónoma de Buenos Aires, Argentina

*jimenacabilla@hotmail.com

^+^These authors contributed equally to this work

**Supplementary Data**

**Western blot raw data:**

- **Figure1**

Anti- sGCα1


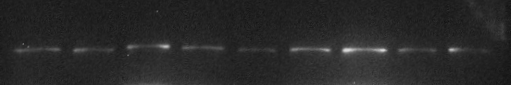
 Chemoluminscent bands

Selected bands


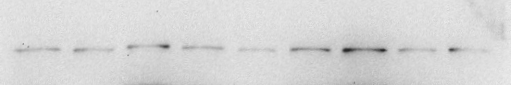
 Inverted bands

Anti- β-actin antibody


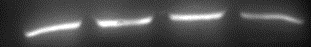
 Chemoluminscent bands


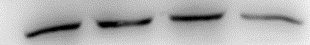
 Inverted bands

- **Figure 3**

Selected bands

Anti-CCD1 antibody


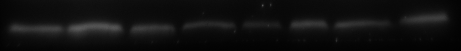
 Chemoluminscent bands


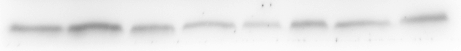
 Inverted bands

Anti-CCNE antibody


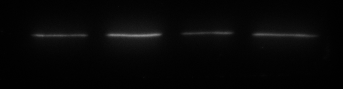
 Chemoluminscent bands


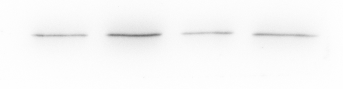
 Inverted bands

Anti-PCNA antibody


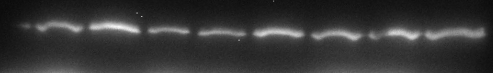
 Chemoluminscent bands


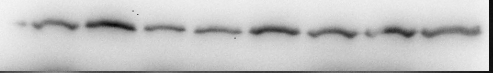
 Inverted bands

Anti- β-actin antibody


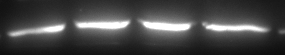
Chemoluminscent bands


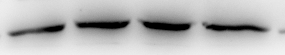
Inverted bands

- **Figure 6**

Anti- sGCα1


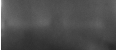
 Chemoluminscent bands


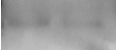
 Inverted bands

Anti-PCNA and Anti-β-actin antibodies


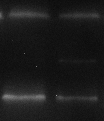

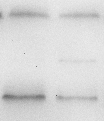


β-actin

PCNA
